# Clinical Outcomes of Rotational Atherectomy in the Drug-Eluting Stent Era

**DOI:** 10.3390/jcm14072199

**Published:** 2025-03-24

**Authors:** Yonghee Kim, Kyusup Lee, Sung-Ho Her

**Affiliations:** 1 Cardiovascular Research Institute for Intractable Disease, College of Medicine, The Catholic University of Korea, Seoul 06591, Republic of Korea; yonghee_89@naver.com (Y.K.); ajobi7121@gmail.com (K.L.); 2 Department of Cardiology, Daejeon St. Mary’s Hospital, College of Medicine, The Catholic University of Korea, Seoul 06591, Republic of Korea; 3 Department of Cardiology, St. Vincent’s Hospital, College of Medicine, The Catholic University of Korea, Seoul 06591, Republic of Korea

**Keywords:** percutaneous coronary intervention, rotational atherectomy, outcomes, indication

## Abstract

**Background**: The increasing prevalence of severe calcified coronary artery disease has expanded the role of rotational atherectomy (RA) in percutaneous coronary intervention (PCI). In the drug-eluting stent (DES) era, RA remains a key tool for complex lesion modification. This review focuses on its clinical outcomes and evolving indications. **Methods**: This review was conducted as a narrative review, focusing on the most relevant clinical studies regarding RA in the DES era. Articles were identified through a systematic PubMed search. **Results**: Comparing to early-generation DES, new-generation DES (NG-DES) demonstrate superior outcomes due to thinner struts and biocompatible polymers. RA plays a critical role in challenging scenarios, including chronic total occlusions and de novo small vessel lesions. Despite these advances, further randomized controlled trials are needed to validate the long-term safety and efficacy of RA-based strategies. **Conclusions**: This review highlights the clinical outcomes of RA in the DES era and its evolving role in contemporary cardiology. RA has shown promising potential for broader clinical applications in complex coronary artery disease. However, critical knowledge gaps remain. Further research is needed to refine RA-based strategies.

## 1. Introduction

Since its introduction in 1977, rotational atherectomy (RA) has been widely used to treat heavily calcified coronary artery disease [1]. Coronary artery calcification (CAC) increases with atherosclerosis, aging, chronic hypercalcemia, hyperlipidemia, or chronic renal disease [2,3]. Indeed, a large scale meta-analysis revealed that the prevalence of moderate to severe CAC was approximately one-third of the overall study population (6211 of 13,622) [4]. With the growing prevalence of calcified lesions in patients, the RA has become increasingly important, highlighting the necessity of understanding its evolving role in tandem with technological advancements [5].

Several plaque modification techniques have been introduced to facilitate percutaneous coronary intervention (PCI) in calcified lesions, including orbital atherectomy and intravascular lithotripsy. However, RA remains one of the most widely utilized techniques due to its distinct mechanism of differential cutting and long-standing clinical experience. This study focuses on RA in the drug-eluting stent era, examining its efficacy and clinical implications.

Recent data from a pooled analysis of the two randomized ROTAXUS and PREPARE-CALC trials showed the technical evolution in RA practice and proposed recommendations to improve RA skills [6]. Those studies demonstrated superior clinical outcomes of new-generation drug-eluting stent (NG-DES) compared to early-generation drug-eluting stent (EG-DES). However, randomized trials often fail to fully capture real-world clinical practice due to inherent selection bias. To address this limitation, we reviewed and summarized clinical outcomes from contemporary studies utilizing real-world data in drug-eluting stent (DES) era.

Within the context, particular attention is given to advancements in the treatment of complex coronary pathologies, such as chronic total occlusion (CTO) and de novo small vessel coronary lesions. Development of RA techniques and device technology have expanded its indications to more complex coronary artery lesions such as CTO [7,8] and de novo small vessel coronary lesions.

Therefore, we focused on (1) clinical outcomes of NG-DES in coronary artery calcification (CAC) compared with EG-DES; (2) studies comparing biodegradable polymer (BP) DES and durable polymer (DP) DES; (3) clinical evidence of RA in chronic total occlusion (CTO) or de novo small vessel coronary lesions.

## 2. Basic Principles of RA

The RA utilizes a metallic burr coated with 20–30 µm diamond crystals. The burr diameter ranges from 1.25 to 2.5 mm. It is performed over a 0.014” guidewire (floppy and extra support) and operates at a high rotational speed of 140,000–180,000 rpm. RA ablates on the principle of differential cutting, selectively applying force to inelastic tissue while sparing the elastic arterial wall. The atheroma component is fragmented into microscopic particles, approximately 5 µm in size, which are smaller than red blood cells, and are cleared via the reticuloendothelial system without causing capillary obstruction. RA enhances lesion preparation by modifying heavily calcified plaques, thereby improving subsequent stent delivery and optimization. However, it carries the risk of complications such as slow flow, perforation, or no-reflow phenomena.

## 3. Methodology

This review was conducted as a narrative review, focusing on the most relevant clinical studies regarding rotational atherectomy in the DES era. Relevant articles were identified through a PubMed database search, restricted to studies published between 2011 and 2024. Keywords were “drug-eluting stent”, “coronary”, “rotational atherectomy”, and “clinical outcome”.

A total of 98 studies were initially retrieved and screened based on predefined inclusion and exclusion criteria. Studies consisted of registry-based studies or retrospective real-world data in DES era and were included if they reported at least 1-year clinical outcomes.

**Figure 1:**
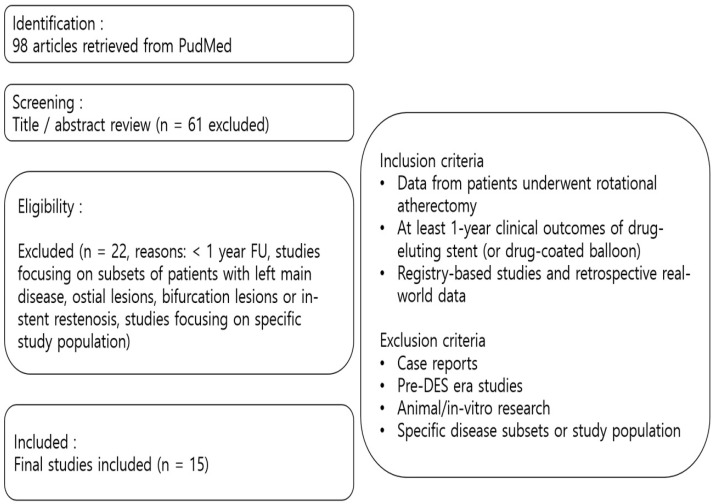
**Flowchart of study selection.** FU, follow-up; DES, drug-eluting stent.

Exclusion criteria included case reports, pre-DES era studies, animal/in vitro research, and studies focusing on specific disease subsets (such as left main disease, ostial lesions, bifurcation lesions or in-stent restenosis) or focusing on specific study population. To ensure clarity in the selection process, a PRISMA-style flowchart (Figure 1) has been provided to summarize the methodology.

## 4. Clinical Outcomes of RA Treated with DES (Early-Generation DES Versus New-Generation DES)

### 4.1. Clinical Impact of CAC During Procedure and on Long-Term Outcomes

Significant CAC is associated with worse clinical outcomes in both the general population and patients undergoing PCI. One of the key reasons is that patients with moderate to severe CAC often had higher chances of comorbidity—such as diabetes mellitus, chronic kidney disease, and hypertension—and more complex CAD [9,10,11,12]. The amount of coronary calcification also correlates with overall atherosclerotic plaque burden [13]. Moreover, microcalcifications within the fibrous cap and calcific nodules have been implicated in recurrent plaque rupture and intraplaque hemorrhage, further exacerbating luminal narrowing and contributing to significant CAD progression and adverse clinical outcomes [14,15].

In addition, CAC introduces several technical challenges during PCI, often complicating the procedural outcomes and increasing the risk of suboptimal outcomes [9]. One of the primary concerns is mechanical resistance, which hinders device delivery [16]. This often necessitates aggressive lesion preparation, including high-pressure non-compliant balloon inflation, cutting or scoring balloons, and atherectomy techniques, to ensure successful advancement of balloons and stents [17,18]. Furthermore, rigid calcified plaques can restrict stent expansion, leading to underexpansion or malapposition, both of which are associated with increased risks of stent thrombosis and in-stent restenosis [4,19,20].

Beyond that, the presence of nodular calcium can increase the risk of vessel injury during procedure, including dissections or perforations, which can be life-threatening. Given these procedural challenges, appropriate lesion preparation and modification through techniques such as RA or intravascular lithotripsy are essential to optimize stent deployment, improve procedural success.

### 4.2. Clinical Performance of EG-DES and NG-DES in CAC Lesions

Guedeney et al. investigated the impact of moderate to severe CAC on long-term clinical outcomes and the respective performance of EG-DES and NG-DES [4]. This meta-analysis of 18 randomized trial demonstrated moderate to severe CAC was independently associated with poorer prognosis. However, patients treated with NG-DES showed significant reduction in the 5-year rates of the patient-oriented clinical endpoint (composite outcome of any death, any revascularization, or any myocardial infarction [MI]) compared with EG-DES.

### 4.3. Clinical Evidences of RA Treated with NG-DES

To reflect real-world practice, we retrospectively analyzed clinical outcomes of RA in the DES era using the ‘Clinical Outcome of Rotational Atherectomy in Calcified Lesions in Korea (ROCK)’ registry. The registry enrolled 540 consecutive patients with severe coronary artery calcification who underwent percutaneous coronary intervention (PCI) using rotational atherectomy (RA) between January 2010 and October 2019 at nine tertiary centers in Korea [21]. In this study, majority of study population (with the exception for one patient) treated with NG-DES, which reflect the clinical outcomes of RA in the NG-DES era. Target vessel failure (TVF, defined as cardiac death, target-vessel spontaneous MI, or target vessel revascularization [TVR]) occurred in 16.0% at 1.5 years, which showed acceptable clinical outcomes.

Several other real-world data of NG-DES were also listed in Table 1. Jinnouchi H et al. reported the cumulative 2-year incidence of major adverse cardiac event (MACE, defined as a composite of cardiac death, MI, clinically driven target lesion revascularization [TLR]) of 20.3% among 252 patients who received NG-DES after RA [22]. Hachinohe D et al. reported that 51 lesions (6.6%) resulted in TVF at 1 year among 744 patients, indicating relatively better clinical outcomes [23]. Okai I et al. reported 25.7% of MACE at 3 years using data from the J2T ROTA registry [5].

There were two studies comparing clinical outcomes of NG-DES versus EG-DES that showed better outcomes of NG-DES (Table 1). Kawamoto H et al. suggested a possible advantage of NG-DES compared to EG-DES (2-year of clinically driven MACE [all-cause death, any MI, and TLR]: 12.9% vs. 19.2%, *p* = 0.04) from subgroup analysis in the ROTATE multicenter registry [24]. Allali A et al. demonstrated that NG-DES showed a lower MACE rate than EG-DES (2 years of MACE [all-cause death, spontaneous MI, and TVR]: 21.1% vs. 31.1%, log-rank *p* = 0.04) [25]. Based on the studies as represented above, the clinical outcomes of NG-DES implantation after RA have been improved compared to those of EG-DES over the past two decades. In addition to drug-eluting device technology, development of optimal strategy of medications, widespread use of intravascular image during procedure, enhanced procedure skills and optimal recommendations of RA, thinner stent strut platforms and more biocompatible polymers of characteristics of NG-DES compared with EG-DES may account for those differences in real-world practice. This review of real-world data highlights the important role of RA, combined with NG-DES, in optimizing clinical outcomes for patients with heavy calcified complex coronary disease.

## 5. Studies Comparing Biodegradable Polymer Versus Durable Polymer DES After RA

### 5.1. Clinical Evidences of BP-DES and DP-DES in CAC Lesions

The novel randomized study, BIO-RESORT trial was conducted by Buiten et al. [26], which was to assess 2-year clinical performance of three thin-strut DES (biodegradable polymer everolimus-eluting stents [BP-EESs] or biodegradable polymer sirolimus-eluting stents [BP-SESs], versus durable polymer zotarolimus-eluting stents [DP-ZESs]) in patients with severely calcified lesions. A total of 783 all-comer patients with severely calcified lesions were assessed for final analysis. Multivariate analysis showed that BP-EESs was independently associated with lower TVR rates compared to DP-ZESs. However, RA was used in 4.4–6.4% of overall patients in the study.

### 5.2. Clinical Evidences of BP-DES and DP-DES After RA

As shown in Table 2, there were two studies comparing biodegradable polymer drug eluting stent (BP-DES) and durable polymer drug eluting stent (DP-DES) after RA. Mankerious et al. reported comparable outcomes between Orsiro biodegradable sirolimus-eluting stent (SES) and durable polymer everolimus-eluting stent (EES) with 285 patients underwent PCI with RA [27]. The rates of target lesion failure (TLF) at 2 years were not significantly different between two groups (BP-DES:18% vs. DP-DES:15%, log-rank *p* = 0.98) However, in a subgroup analysis with small-sized (≤3.0 mm) stent group, the incidence rate of TLF was significantly lower in the BP-DES group than that of DP-DES group (3% vs. 19%, log-rank *p* = 0.004), mainly driven by TLR (2% vs. 12%, log-rank *p* = 0.022). Because of inherent limitations of study design (single-center, retrospective non-randomized) and the small size of the study population, generalization of the results should be performed with caution.

Recently, Kim et al. aimed to demonstrate the better outcomes of BP-DES compared to DP-DES in patients underwent RA with larger study population (n = 510, Table 2) [28]. BP-DES was associated with lower incidence rates of all-cause mortality and cardiovascular mortality compared to DP-DES (4.7% vs. 10.7%, hazard ratio [HR] 0.41, *p* = 0.047; 3.4% vs. 8.1%, HR 0.28, *p* = 0.024, respectively). However, due to higher incidence rates of TVR or TLR in BP-DES than DP-DES, TVF did not show any significant differences between the two groups. Because both studies were designed retrospectively, those different results of the previous studies were enough to explain the need for further randomized study regarding comparison of clinical outcomes of BP-DES versus DP-DES in patients treated with RA.

## 6. Clinical Impact of RA in CTO

### 6.1. Case

**Figure 2:**
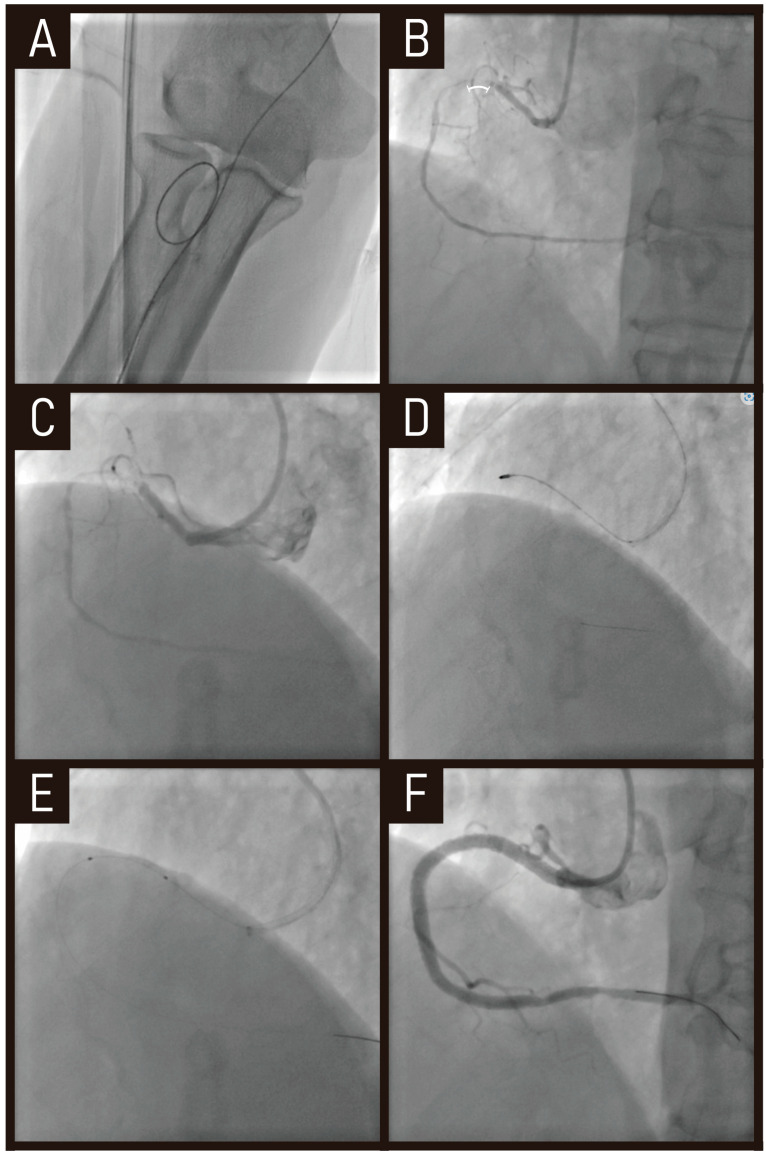
**Case of RA in CTO-PCI.** RA with a 1.25 mm burr was performed to modify a calcified CTO lesion, allowing procedural success in this previously failed case. (**A**) Severely tortuous brachial artery. (**B**) CTO segment (marked by a white bracket). (**C**) Ikari right 1.5 guiding catheter providing better engagement and stability. (**D**) RA with a 1.25 mm burr performed in the CTO lesion. (**E**,**F**) balloon inflation and stent implantation. RA, rotational atherectomy; CTO-PCI, chronic total occlusion-percutaneous coronary intervention.

A 78-year old presented with stable angina. Two months prior, he underwent an unsuccessful CTO-PCI for proximal right coronary artery CTO. Despite attempts using the anchoring balloon technique with a Judkins right 4.0 guiding catheter, the balloon failed to cross the lesion. Right coronary angiogram showed short CTO segment and microchannel formation from the previous intervention (Figure 2B). However, an additional challenge was the severely tortuous brachial artery, which resulted in poor back-up support with a conventional guiding catheter (Figure 2A).

To overcome these limitations, we utilized an Ikari right 1.5 guiding catheter, which provided better engagement and stability (Figure 2C). Additionally, RA with a 1.25 mm burr was performed to modify the calcified lesion (Figure 2D), allowing for successful balloon inflation and stent implantation (Figure 2E,F).

This case highlights how RA, in combination with an optimized guiding catheter selection, can overcome anatomical challenges such as tortuous access and poor back-up support, ultimately enabling successful PCI in complex CTO lesions.

### 6.2. Prevalence of Calcification in CTO Lesions

Calcification of CTOs are more prevalent in those of longer occlusion duration [29]. On intravascular ultrasound (IVUS) analysis, Guo et al. reported that 64% of CTOs had predominantly fibrocalcific plaque [30]. A moderate or severe calcification was present in 58% of CTO lesions as assessed by angiography [31]. From the PROGRESS registry, Japanese Multicentre registry reported 57–59% of CTOs had angiographically moderate or severe calcification [32,33].

Calcification in CTO lesions is an independent predictor of lower procedural success rates or higher risk of complications [32,34,35]. The presence of calcium at the proximal cap can pose a significant hurdle to the passage of wires or microcatheters, sometimes resulting in deflection into the subintimal space [36]. After successful device crossing, adequate lesion preparation and the modification of calcified plaques are crucial steps prior to stent implantation to ensure stent optimization and better outcomes [37,38]. However, heavily calcific plaques act as a challenge due to their refractory characteristics and rough lesion surface. This can contribute the failure of stent delivery or insufficient stent expansion [39,40,41,42].

### 6.3. Clinical Usefullness of RA in CTO Lesions

RA has been considered to be the effective procedure in CTO PCI and might enhance the procedure success and the preparation of calcified lesions [43,44]. With debulking the atheromas, RA can enable balloon dilatation, plaque fracture, stent delivery, and expansion [45]. Also, the utilization of RA facilitates a minimally invasive strategy by allowing interventions that typically require a large bore catheter via the femoral approach to be performed through a radial approach with a 6 Fr guiding catheter. This adaptation may benefit patients by reducing procedural invasiveness and the associated risks.

### 6.4. Clinical Outcomes of RA in CTO Lesions

We demonstrated the usefulness of RA in chronic total occlusion (CTO) lesions compared to non-CTO lesions. Lee et al. reported the feasible success rates of RA in CTO-PCI (90.5 % in CTO-PCI group vs. 96.9 % in non-CTO-PCI group, *p* = 0.06) and clinical outcomes compared to the non CTO-PCI group (Table 3) [46]. Although 42 CTO lesions were enrolled, which were relatively small study population, any MI or stent thrombosis (ST) did not occur. This finding could be interpolated in our clinical practice for operators adjusting RA during CTO-PCI. Another important finding is higher proportion of IVUS use in CTO-PCI group than non-CTO-PCI group (69.1 % vs. 43.8 %, *p* = 0.002, respectively).

Another study in the literature showed acceptable long-term clinical outcomes of RA-PCI compared with conventional PCI for CTO. Ayoub et al. reported that RA-PCI had significantly better procedural success rates compared to those treated without RA (93.3% vs. 85.1%, *p* = 0.0002). Although the 1-year MACCE rate was similar in both groups (18.7% vs. 16.7%, *p* = 0.49) [47], Huang et al. demonstrated better clinical outcomes of a RA-PCI group over a period of more than 4 years compared to a non-RA-PCI group. In the DES subgroup, long-term MACE, non-fatal MI, and TLR were similar across both groups. However, regarding CV death, RA-PCI showed significant fewer events [8]. Azzalini et al. reported procedural success rates reached by 77% in RA for CTO PCI. During a mean follow-up duration of 658 ± 423 days, the incidence rates of primary endpoint (MACE) were similar between two groups (15% vs. 13%, *p* = 0.70, Table 3) [48]. In RA group, even 20% was in the setting of dissection/re-entry; thus, no perforation occurred. In an editorial comment, Brilakis et al. emphasized that RA is an important tool for complex CTO-PCI, especially in cases of balloon uncrossable and undilatable lesions, and in such case of dissection/re-entry, subintimal RA may be safe in CTO-PCI [49]. Recently, meta-analysis including seven studies with 5494 patients reported that RA was comparable to conventional percutaneous coronary intervention (PCI) without RA for CTO lesions [50].

## 7. RA Co-Treated with Drug-Coated Balloon

### 7.1. Case

**Figure 3:**
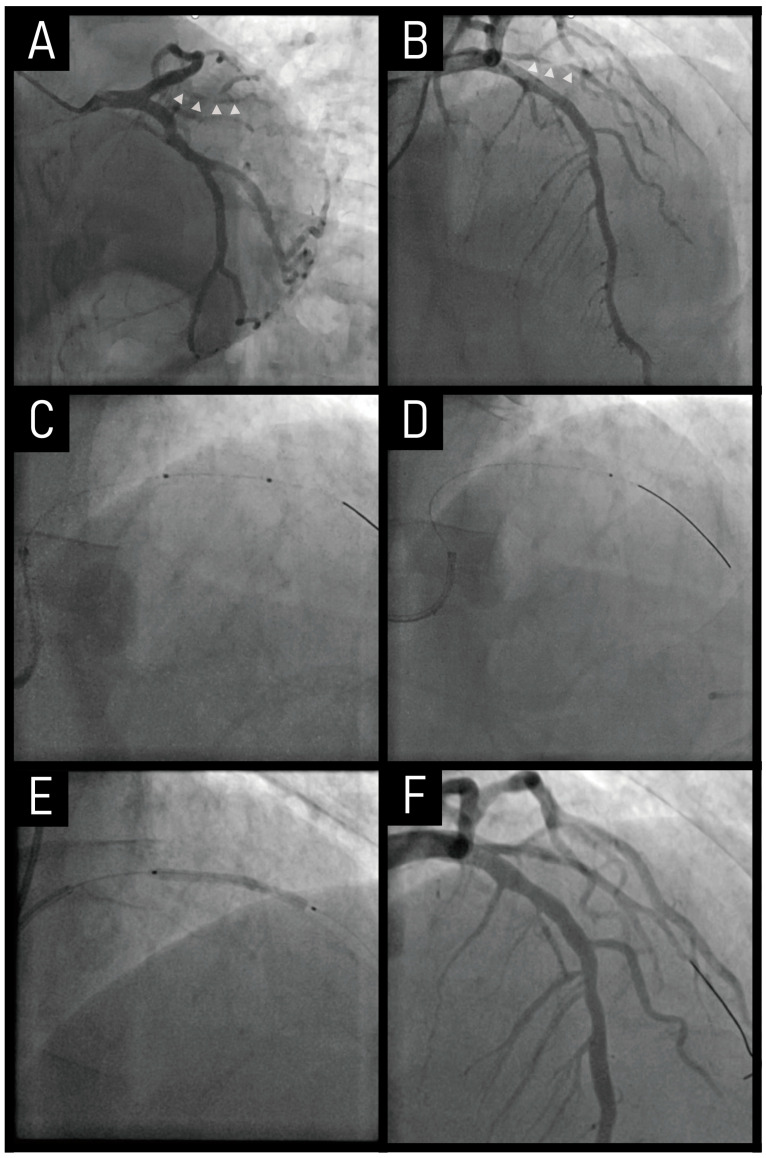
**Case of RA co-treated with a drug-coated balloon.** The combination of RA with DCB therapy facilitates plaque modification and debulking, providing an alternative to stent implantation in small vessel disease. (**A**,**B**) Severe stenosis in the first diagonal branch (marked by small white triangles). (**C**) A 2.0 mm plain balloon failed to cross the lesion. (**D**) A 1.5 mm plain balloon was used. (**E**) A DCB angioplasty was performed. (**F**) The final angiogram showed a good result. RA, rotational atherectomy; DCB, drug-coated balloon.

A 63-year old male presented with a one-week history of resting chest pain. His past medical history was treated hypertension. Invasive coronary angiography revealed a long-segment, severe (99%) stenosis in the first diagonal branch (Figure 3A,B). An initial attempt with a 2.0 mm plain balloon failed to cross the lesion (Figure 3C). Even after inflating a 1.5 mm plain balloon, the Finecross microcatheter was still unable to advance across the lesion (Figure 3D).

To overcome this challenge, a 1.25 mm burr of RA was performed, followed by a DCB angioplasty, both of which were successfully completed in sequence (Figure 3E,F). A critical technical difficulty arose when the rota wire failed to advance to the distal vessel. To address this, the wire was gently maneuvered under Dynaglide mode, allowing for its correct positioning in the distal segment. This precise wire manipulation facilitated safe execution of RA, ensuring optimal lesion preparation without compromising vessel integrity.

This case highlights that, despite the inherent risks of RA in small vessels, it can be selectively utilized in refractory lesions where conventional approaches fail. Additionally, the combination of RA with DCB therapy may offer a promising strategy to improve long-term outcomes by facilitating plaque modification and debulking, providing an alternative to stent implantation, and reducing restenosis risk in such small vessel disease.

### 7.2. Clinical Evidences of Drug-Coated Balloon in De Novo Small Coronary Vessels

DCB has been shown to be safe and effective for use in de novo small coronary vessels [51]. To date, there have been several DCB studies compared with DES in de novo small vessel coronary lesions. In BELLO randomized controlled trial, DCB was associated with better angiographic outcomes and similar rates of adverse events as compared with first-generation DES [52]. After 3 years, a better clinical outcome emerges for DCB, in terms of MACE [53]. Two additional studies demonstrated non-inferiority of DCB compared with DES in terms of angiographic and clinical outcomes [54,55]. More recently, in the PICCOLETO randomized controlled trial, they showed the angiographic superiority in terms of late lumen loss of DCB over second-generation DES [56]. There have also been some studies that showed acceptable outcomes of DCBs in calcified coronary lesions [57,58]. However, it is unclear whether DCBs are effective in calcified coronary lesions.

### 7.3. Clinical Outcomes of RA Co-Treated with Drug-Coated Balloon in De Novo Small Vessel Coronary Lesions

Building on this background, clinical experiences of RA co-treated with DCB have been accumulated and translated in the literature as follows (Table 4). Nagai reported a single-center data of the acute and mid-term efficacy of DCB following RA. At mid-term follow-up duration (196 ± 37 days), there were two deaths (non-cardiovascular death), 16.4% of TLR, and 20.7% of TVR [59]. Dong et al. demonstrated safe and effective results of DCB-RA compared with DES-RA in selected severe coronary artery calcification [60]. There were similar incidence rates of major procedural complications and in-hospital events. DCB-RA group showed numerically lower incidence rates of TLR (13.8% vs. 7.0%) and MACCE (18.8% vs. 12.3%) compared with DES-RA group without statistical significance.

## 8. Discussion and Future Direction

We reviewed and summarized clinical outcomes of RA treated with DES, with a particular focus on NG-DES. Clinical evidence consistently demonstrated the superior outcomes of NG-DES compared to EG-DES, even in severe CAC lesion treated with RA. The reported incidence rates of MACE (or TVF) ranged from 6.6% at 1 year to 21.1% at 1.5 years in NG-DES groups [21,22,23,61]. The observed improvements in clinical outcomes may be attributed to advancements in stent design, such as thinner strut platforms and more biocompatible polymers [62,63]. However, reviewed studies have inherent limitations that warrant careful interpretation. Notably, study periods for three of the five studies listed in Table 1 spanned approximately a decade, which may not fully reflect current clinical practices. Moreover, most of these studies were conducted before 2015, limiting their ability to incorporate recent innovations in medication strategies (including potent P2Y12 inhibitors, high-intensity statins, PCSK9 inhibitors, and four pillars of therapy for heart failure with reduced ejection fraction) and the concomitant use of intravascular imaging during procedures. Especially, intravascular imaging guidance is recommended by the European expert consensus on RA for ACS patients undergoing PCI with a Class IIA recommendation [64,65]. In CCS patients for complex lesions (such as heavily calcified ones), the most recent CCS guidelines assigned a Class I recommendation [66]. Intravascular imaging modalities, including IVUS and optical coherence tomography, offer complementary morphological and quantitative assessment of calcification and facilitate the optimal selection of techniques and devices during procedures [67]. However, as shown in our review, real-world data indicate that intravascular imaging is often underutilized (31.2–99.6%). Among these studies, a particularly noteworthy one is the recent study by Hachinohe et al. [23], in which intravascular imaging was used in 99.6% of patients. Interestingly, this study reported relatively better clinical outcomes compared to others. While the high utilization of intravascular imaging may have contributed to these results. Despite guideline recommendations, its utilization in real-world practice remains suboptimal, highlighting the need for improved implementation strategies.

Recently, the development of ultra-thin coronary stent (struts thickness < 70 μm) has emerged as a promising advancement in PCI. These stents, with their thinner struts, offer improved deliverability and reduced vessel injury, which may optimize outcomes, particularly in complex coronary lesions. Studies comparing ultra-thin stents with thicker-strut NG-DES have demonstrated non-inferiority in terms of TLF [68,69]. Consequently, ultra-thin coronary stents are now widely adopted in clinical practice due to their improved deliverability and reduced vessel injury, which may optimize outcomes in complex coronary lesions [70,71,72,73]. However, their performance in heavily calcified lesions remains insufficiently studied, highlighting the need for further research in this area.

In summary, limitations of the reviewed studies include prolonged study durations and the underrepresentation of ultra-thin coronary stents, underscoring the need for further research. Large-scale registries or randomized controlled trials are warranted to comprehensively evaluate the clinical outcomes of RA performed with ultra-thin coronary stents. These studies should integrate contemporary medication strategies as mentioned above as well as the mandatory use of intravascular imaging, to ensure that findings align with current clinical practices and provide robust evidence for optimal procedural strategies in heavily calcified lesions.

We also reviewed two studies comparing clinical outcomes based on polymer types, BP-DES and DP-DES. As summarized in Table 2, BP-DES showed a trend toward lower incidence rates of primary endpoint, although this did not reach statistical significance. Additionally, two studies reported conflicting results regarding TLR rates, highlighting potential variability in outcomes. However, in a subgroup analysis with small-stent groups (≤3.0 mm) of one study [27], BP-DES demonstrated better clinical outcomes than DP-DES, particularly in terms of cardiac death, MI, and TLR. These findings suggest that while BP-DES may offer certain advantages in subgroups with heavily calcified small vessel disease, the lack of consistent results underscores the need for further investigations. Further randomized controlled trials with larger cohorts and robust methodologies are urgently needed to validate these observations and provide practical insights into the optimal use of BP-DES versus DP-DES in heavily calcified coronary lesions. Specifically, future randomized controlled trials should address the limitations of prior research by incorporating stratification based on stent diameter (e.g., <3.0 mm vs. ≥3.0 mm), enabling a clearer understanding of the comparative performance of these stents in different lesion and vessel characteristics. Such an approach would not only address variability in outcomes but also refine procedural strategies for patients with heavily calcified small vessel disease.

We presented a case of CTO-PCI using RA, in a patient with poor co-axial and back-up support from the guiding catheter due to anatomical challenges. The procedure initially failed but was successfully performed using a 1.25 mm burr RA followed by balloon dilatation. According to current guideline [45], a single run with a 1.25 mm burr RA, as in our case, is generally sufficient to achieve good plaque modification in most cases. Given the clinical significance of RA in CTO-PCI, we also reviewed two studies investigating its clinical outcomes in this setting. One study demonstrated clinical efficacy of RA in CTO-PCI group compared to non-CTO-PCI group. As mentioned above, IVUS was more frequently used in CTO-PCI group than non-CTO-PCI group. Despite the inherent limitations of selection bias due to the small study population, these findings suggest that potential benefits of combining RA with IVUS in CTO-PCI to achieve acceptable and feasible clinical outcomes. However, in this study, patients who underwent RA were retrospectively compared between CTO-PCI and non-CTO-PCI groups. Because lesion characteristics were significantly different in terms of plaque burden, lesion length and the density of calcification, comparing two groups would not be appropriate. To overcome these limitations and accurately assess the efficacy of RA, it is crucial to design future studies with more refined methodologies. Specifically, future research should focus on conducting randomized controlled trials comparing combined RA + IVUS strategies with IVUS-only approaches in CTO-PCI procedures. This design would enable a direct evaluation of the additive benefits of RA. Such trials would provide reliable evidence to guide clinical decision-making and optimize procedural strategies for CTO-PCI.

We also explored the safety of RA in the case of dissection/re-entry during CTO-PCI. While some experts have suggested that subintimal RA may be a safe option in CTO-PCI, this remains a topic of ongoing debate. Although the literature we presented suggests the potential safety of RA, concerns about coronary dissection or perforation during RA in CTO-PCI remain significant. Thus, the decision to perform subintimal RA should be made cautiously and on a case-by-case basis by the operator, considering the individual patient’s risk profile and lesion characteristics. Expert consensus suggests that in heavily calcified CTOs where the lesion is crossed via subintimal tracking, balloon inflation pressures exceeding 14–16 atm and atherectomy are not advised [67].

In this paper, two studies of RA co-treated with DCB have been introduced. RA facilitates treatment by modifying calcified lesions, while DCB reduces the risk of restenosis through drug elution without the need for stent implantation. This combination presents a potential therapeutic option for the management of small vessel diseases. However, concerns remain regarding the risk of vascular injury caused by RA in small-caliber vessels and the uncertain efficacy of drug delivery from DCB in heavily calcified lesions.

Additionally, calcified nodules may increase the risk of adverse events or coronary perforation even after stent implantation [74]. In such cases, the combination of calcium modification techniques, such as atherectomy or intravascular lithotripsy, followed by DCB treatment, may serve as an optimal strategy for managing calcified nodule. However, evidence supporting the efficacy and safety of this strategy remains limited, and further research is needed to establish its clinical utility in this specific setting.

Nevertheless, in patients at a high bleeding risk, the ability to avoid stent implantation and minimize the duration of dual antiplatelet therapy while achieving effective revascularization suggests that this approach could be both practical and valuable when safely applied to selected patient populations. Further research is needed to validate the efficacy and safety of this strategy and to establish its potential for broader clinical applications.

### Limitations

One of the key challenges in interpreting the available literature from our study is the heterogeneity observed across studies. This heterogeneity arises from differences in study design (prospectively enrolled registry-based studies vs. retrospective studies), study population, lesion characteristics, and procedural strategies. Additionally, variations in clinical endpoints, definitions of MACE, and follow-up durations further contribute to the difficulty in making direct comparisons between studies.

As a result, the reported clinical outcomes of RA in contemporary practice show considerable variation, making it difficult to derive definitive conclusions regarding its efficacy and safety outcomes. While randomized trials provide robust data, real-world registries offer valuable insights into clinical practice patterns. However, the inherent limitations of observational studies, including selection bias and the presence of unmeasured confounders, must be considered.

Future studies should aim to standardize study population and definitions of outcome to facilitate more meaningful comparisons. Furthermore, subgroup analyses focusing on lesion subsets, the role of intravascular imaging, and post-procedural pharmacotherapy may provide deeper insights into optimizing RA strategies in contemporary clinical practice.

## 9. Conclusions

RA has demonstrated favorable clinical outcomes, particularly when used with NG-DES. Clinical evidence consistently demonstrated the superior outcomes of NG-DES compared to EG-DES, even in severe calcified lesions treated with RA.

However, several critical gaps in knowledge remain, highlighting the need for future investigations. First, while ultra-thin coronary stents have shown promising results, their performance in heavily calcified lesions remains insufficiently studied. Large-scale registries and randomized controlled trials (RCTs) are needed to assess their efficacy in this subset. Second, the comparative effectiveness of BP-DES versus DP-DES in heavily calcified small vessel disease remains unclear. Further RCTs incorporating stratified analyses based on stent diameter could provide clearer insights into their clinical benefits.

Additionally, the role of RA in CTO-PCI warrants further investigation. Future studies should evaluate whether the addition of RA to intravascular imaging improves procedural and long-term outcomes compared to IVUS-guided PCI alone. Finally, while DCBs following RA offer a potential strategy to reduce restenosis in calcified nodules and small vessel disease, particularly in patients who are unsuitable for DES implantation.

## Figures and Tables

**Table 1:** Comparison of clinical outcomes according to early generation DES versus new generation DES after RA.

	Study Population	Era	Duration	Study Period	IVUS or OCT, %	MACE, %	All-Cause Death, %	MI, %	TVR, %	TLR, %	ST, %
Lee et al. [21]	540	NG-DES	1.5 years	2010–2019	46.0	16.0 *	8.4	2.1	9.8	8.2	1.2
Kawamoto et al. [24]	985	8.8% BMS	2 years	2002–2013	31.2	32.2 **	9.5	3.3	19.8	16.6	1.8
		21.9% EG-DES				19.2 **					
		69.3% NG-DES				12.9 **					
Allali et al. [25]	268	55.7% EG-DES	2.5 years	2002–2015	NA	31.1	13.5	4.9	17.6	12.7	0.9
		44.3% NG-DES	1.5 years			21.1	8.2	4.1	12.9	7.9	2.4
Jinnouchi et al. [22]	252	NG-DES	2 years	2010–2012	NA	20.3	13.5	2.1	24.8	21.9	2.1
Hachinohe et al. [23]	744	NG-DES	1 year	2013–2015	99.6	6.6 *	5.5	0.1	-	2.9	0.1
Okai et al. [5]	1090	11.2% BMS	3.8 years	2004–2015	73.0	45.5	24.2	6.8 ^$^	21.4	16.2	1.3
		52.5% EG-DES									
		36.3% NG-DES									

* target vessel failure defined as cardiac death, target-vessel spontaneous MI, and TVR; ** clinically driven MACEs; ^$^ acute coronary syndrome instead of MI. DES, drug-eluting stent; RA, rotational atherectomy; IVUS, intravascular ultrasound; OCT, optical coherence tomography; MACE, major adverse cardiac events; MI, myocardial infarction; TVR, target-vessel revascularization; TLR, target-lesion revascularization; ST, stent thrombosis; CVA, cerebrovascular accident; NG, new-generation; EG, early-generation; BMS, bare metal stent.

**Table 2:** Comparison of clinical outcomes according to biodegradable polymer versus durable polymer DES after RA.

	Study Population	Stent Type	Duration	Study Period	Primary Endpoint, %	All-Cause Death, %	Cardiac Death, %	MI, %	TVR, %	TLR, %	ST, %
Mankerious et al. [27]	285	42.5% BP-DES	2 years	2007–2018	10.0 *	-	5.0	1.0	-	4.0	0
		57.5% DP-DES			18.0 *	-	9.0	2.0	-	10.0	2.0
Subgroup analysis with small-stent group [27]	168	40.5% BP-DES	2 years	2007–2018	3.0	-	2.0	0	-	2.0	-
		59.5% DP-DES			19.0	-	8.0	2.0	-	12.0	-
Kim et al. [28]	510	46.7% BP-DES	3 years	2010–2019	12.2 **	4.7	3.4	1.3	10.2	8.5	1.3
		53.3% DP-DES			13.6 **	10.7	8.1	1.9	6.7	5.9	0.7

* target lesion failure defined as cardiac death, target-vessel MI, and TLR; ** target vessel failure defined as cardiac death, target-vessel spontaneous MI, and TVR; DES, drug-eluting stent; RA, rotational atherectomy; MI, myocardial infarction; TVR, target-vessel revascularization; TLR, target-lesion revascularization; ST, stent thrombosis; CVA, cerebrovascular accident; NG, new-generation; EG, early-generation; BMS, bare metal stent.

**Table 3:** Clinical outcomes of RA in CTO-PCI.

	Patients	Primary Endpoint, %	All-Cause Death, %	Cardiac Death, %	MI, %	TVR, %	TLR, %	ST, %
Lee et al. [46]	CTO, N = 42	14.3 *	4.8	4.8	0	9.5	-	0
	Non-CTO, N = 541	12.9 *	7.8	5.7	3.3	7.0	-	1.3
Ayoub et al. [47]	RA, N = 193	18.7 **	5.7	-	1.0	18.7	18.1	-
	Without RA, N = 2596	16.7 **	3.7	-	1.2	16.3	14.3	-
Huang et al. [8]	RA, N = 25	12.0 *	-	4.0	8.0	-	4.0	-
	Without RA, N = 205	19.5 *	-	11.7	9.8	-	8.3	-
Azzalini et al. [48]	RA, N = 35	15.0 *	-	6	9	6	-	-
	Without RA, N = 968	13.0 *	-	3	3	9	-	-

* target lesion failure defined as cardiac death, target-vessel MI, and TVR; ** major adverse cardiovascular and cerebral events defined as all-cause death, MI, stroke, and TVR; RA, rotational atherectomy; CTO, chronic total occlusion; MI, myocardial infarction; TVR, target-vessel revascularization; TLR, target-lesion revascularization; ST, stent thrombosis.

**Table 4:** Clinical outcomes of DCB after RA.

	**Patients**	**Primary Endpoint, %**	**All-Cause Death, %**	**Cardiac Death, %**	**MI, %**	**TVR, %**	**TLR, %**	**ST, %**
Nagai et al. [59]	167	-	2	0	-	20.7	16.4	-
Dong et al. [60]	DCB-RA, N = 57	12.3 *	1.8	-	0	-	7.0	-
	DES-RA, N = 261	18.8 *	1.5	-	1.2	-	13.8	-

* major adverse cardiovascular and cerebrovascular events defined as all-cause death, non-fatal MI, TLR and stroke; RA, rotational atherectomy; CTO, chronic total occlusion; MI, myocardial infarction; TVR, target-vessel revascularization; TLR, target-lesion revascularization; ST, stent thrombosis.

## Data Availability

No new data were created or analyzed in this study. Data sharing is not applicable to this article.

## Abbreviations

CAD	coronary artery disease
DES	drug-eluting stent(s)
MACE	major adverse cardiac event
MI	myocardial infarction
PCI	percutaneous coronary intervention
RA	rotational atherectomy
TVF	target-vessel failure
TVR	target-vessel revascularization
